# Effect of silicone oil on peripapillary capillary density in patients with rhegmatogenous retinal detachment

**DOI:** 10.1186/s12886-020-01533-7

**Published:** 2020-07-07

**Authors:** Erqian Wang, Youxin Chen, Ningning Li, Hanyi Min

**Affiliations:** 1 Department of Ophthalmology, Peking Union Medical College Hospital, No.1 Shuai Fu Yuan, Dongcheng District, Beijing, 100730 China; 2 Key Laboratory of Ocular Fundus Diseases, Chinese Academy of Medical Sciences, Beijing, 100730 China; 3 Department of Operating Room, Peking Union Medical College Hospital, Beijing, 100730 China

**Keywords:** Radial peripapillary capillaries, Silicone oil, Optic neuropathy, Rhegmatogenous retinal detachment, Optical coherence tomography angiography

## Abstract

**Purpose:**

To evaluate the effect of silicone oil (SO) on peripapillary blood flow using OCT angiography.

**Methods:**

This prospective case series recruited patients with unilateral rhegmatogenous retinal detachment (RRD) who underwent vitrectomy and SO tamponade. Patients were examined before SO removal and at 10 days, 1 month, and 3 months after SO removal on a spectral domain OCT angiography device (RTVue XR Avanti, Optovue Inc., CA, USA) for the measurement of radial peripapillary capillaries (RPC) vessel density (VD) in global field, superior hemifield, and inferior hemifield. Changes in RPC VD following SO removal were compared between affected eyes and contralateral eyes.

**Results:**

Twenty-two patients were analyzed. The average duration of SO tamponade was 101.3 days (range, 90 to 119 days). After SO removal, global RPC VD increased by 1.3% (95%CI, 0.3 to 2.3%), compared with a − 0.4% (95%CI, − 1.4 to 0.7%) change in contralateral eyes (*P* = 0.007). The increase in RPC VD after SO removal mainly occurred in the superior hemifield, which was 1.6% (95%CI, 0.6 to 2.7%). The increase in RPC VD in the inferior hemifield was 1.0% (95%CI, − 0.2 to 2.1%) after SO removal.

**Conclusions:**

We detected a mild increase in peripapillary capillary density after SO removal, mainly in the superior hemifield. Our results suggested that SO tamponade could have negative effect on peripapillary blood flow, possibly by capillary compression.

## Background

Silicone oil (SO) tamponade, widely used in vitreoretinal surgery, is known to be associated with various complications including cataract, keratopathy, glaucoma, and optic neuropathy [1–3]. Optic neuropathy following SO tamponade, subclinical or visually significant, is reported to occur at an incidence rate varying from 0 to 28% [2, 4]. The pathogenesis of SO related optic neuropathy remains unclear. Possible mechanisms may include high intraocular pressure (IOP) [2] and migration of microdroplets of SO into optic nerve [3, 5]. In patients who underwent vitrectomy and SO tamponade for rhegmatogenous retinal detachment (RRD) repair, optic coherence tomography (OCT) revealed retinal thickness changes, particularly in the retinal nerve fiber layers (RNFL) [6–10]. Despite the extensive studies on structural changes in optic nerve, the effect of SO on blood flow around optic nerve has not yet been studied.

Radial peripapillary capillaries (RPC), the innermost layer of capillaries that runs parallel to peripapillary RNFL, function to nourish RNFL in its distribution around the optic nerve head. With the advent of new imaging modality like OCT angiography, in vivo imaging of RPC becomes feasible [11], and quantitative evaluation of RPC is also available. Reduced or abnormal blood flow in RPC has been described in glaucoma and other optic nerve diseases [12–14].

Among the various conditions which need vitreoretinal surgery and SO tamponade, RRD serves as an ideal model to study the effect of SO on RPC, because the optic nerve head at presentation can be absence of intrinsic vascular abnormalities. Also, during the vitreoretinal surgery and SO removal surgery, internal limiting membrane peeling is not always necessary, therefore the likelihood of developing disassociated optic nerve fiber layer appearance [15, 16] or other retinal structural changes [17] is minimized. We thereby enrolled a prospective cohort of patients who underwent successful vitrectomy for RRD repair and subsequent SO removal, and measured RPC vessel density (VD) with SO and after SO removal. We hypothesized that SO filled eyes have transient decreased blood flow in RPC which will recover after SO removal. The purpose of the study was to evaluate the effect of SO on peripapillary vessel density.

## Methods

This prospective, consecutive cohort study was conducted at Ophthalmology Department, Peking Union Medical College Hospital (PUMCH). This study adhered to the tenets of the Declaration of Helsinki. Ethics committee of PUMCH approved the study protocol. All participants provided written informed consent.

### Subjects and clinical protocols

Patients with unilateral rhegmatogenous retinal detachment who underwent uncomplicated pars plana vitrectomy (PPV) and SO tamponade were enrolled from June 2018 to February 2019. Patients were not considered for enrollment if they had any of the following condition in either eye at presentation: 1) high myopia of over − 6.00 diopters or axial length (AL) more than 26.50 mm; 2) history of glaucoma, ischemic optic neuropathy, uveitis, or other retinal or optic nerve diseases; or 3) history of ocular surgery except for refractive and cataract surgery or 4) systemic diseases except for controlled hypertension. Patients had SO removal at least 3 months after primary vitrectomy and had follow-up visits at 10 days, 1 month, and 3 months after SO removal. Patients were excluded from analysis if they: 1) failed to complete follow-up; 2) had recurrent retinal detachment which needed secondary surgical repair; 3) had SO removal combined with epiretinal membrane peeling or internal limiting membrane peeling which might cause iatrogenic RNFL and RPC changes; or 4) had insufficient OCT angiography signal strength (scan quality index < 6/10) due to severe cataract at any follow-up visit.

We also collected the following clinical characteristics: 1) macula status at presentation; 2) whether cataract surgery was performed in combined with SO removal; 3) duration of SO tamponade; and 4) whether SO tamponade was full indicated by whether SO-water interface was invisible under indirect ophthalmoscope after pupil dilation.

### Surgical techniques

All patients had standard 25G PPV and SO tamponade under retrobulbar anesthesia. One single surgeon (H.M.) performed all surgical procedures using Constellation Vision System (Alcon Laboratories, Inc., Texas, USA) and 25G trocar cannula system. The IOP was set at 25 mmHg during surgery. Retinal detachment was repaired in a standard fashion. Briefly, following vitreous removal, vitreoretinal traction release, subretinal fluid drainage and endolaser retinopexy of the retinal breaks, silicone oil (Oxane 5700, Bausch & Lomb, Rochester, N.Y., USA) was instilled. In all patients, the retina was completely reattached at the end of surgery. At the primary vitrectomy surgery, we did not perform combined cataract surgery because none of the study subjects had visually significant cataract. The major concern of performing cataract surgery at primary surgery was that the gradual opacification of lens capsule at the capsulorhexis margin might interfere with adequate peripheral retina examination before SO removal. Another concern was that removing cataract might increase the risk of SO migration into the anterior chamber in eyes with zonular weakness. Prone position was adopted within 2 weeks after vitrectomy. Following vitrectomy surgeries, patients used topical antibiotics, corticosteroid, and IOP-lowering medications in case of need.

The same surgeon (H.M.) performed SO removal surgeries using Constellation Vision System and 23G trocar cannula system, with IOP set at 25 mmHg. Fluid-air exchange was done to eliminate residual SO droplets. Cataract surgery was performed in phakic eyes of patients over 60 years old and in eyes with visually significant cataract. In these patients, phacoemulsification cataract removal was performed before silicone oil removal, and intraocular lens was implanted after silicone oil removal. Scleral wound was sutured in all patients. Topical antibiotics and corticosteroid were applied and tapered off within 4 weeks after SO removal.

### OCT angiography imaging and processing

For optic disk OCT angiography scan we used a commercially available spectral domain OCT angiography device RTVue XR Avanti (Optovue Inc. Fremont, CA, USA). This device uses light source of 840 nm and operates at scan speed of 70,000 A-scans per second. Both the affected eye and the healthy contralateral eye were imaged on a 4.5 mm × 4.5 mm optic disc scan via dilated pupil. Two consecutive scans were performed and the one with optimal image quality and without motion artifacts were used for analysis. All image acquisitions were made by one single investigator (E.W.).

**Fig. 1:**
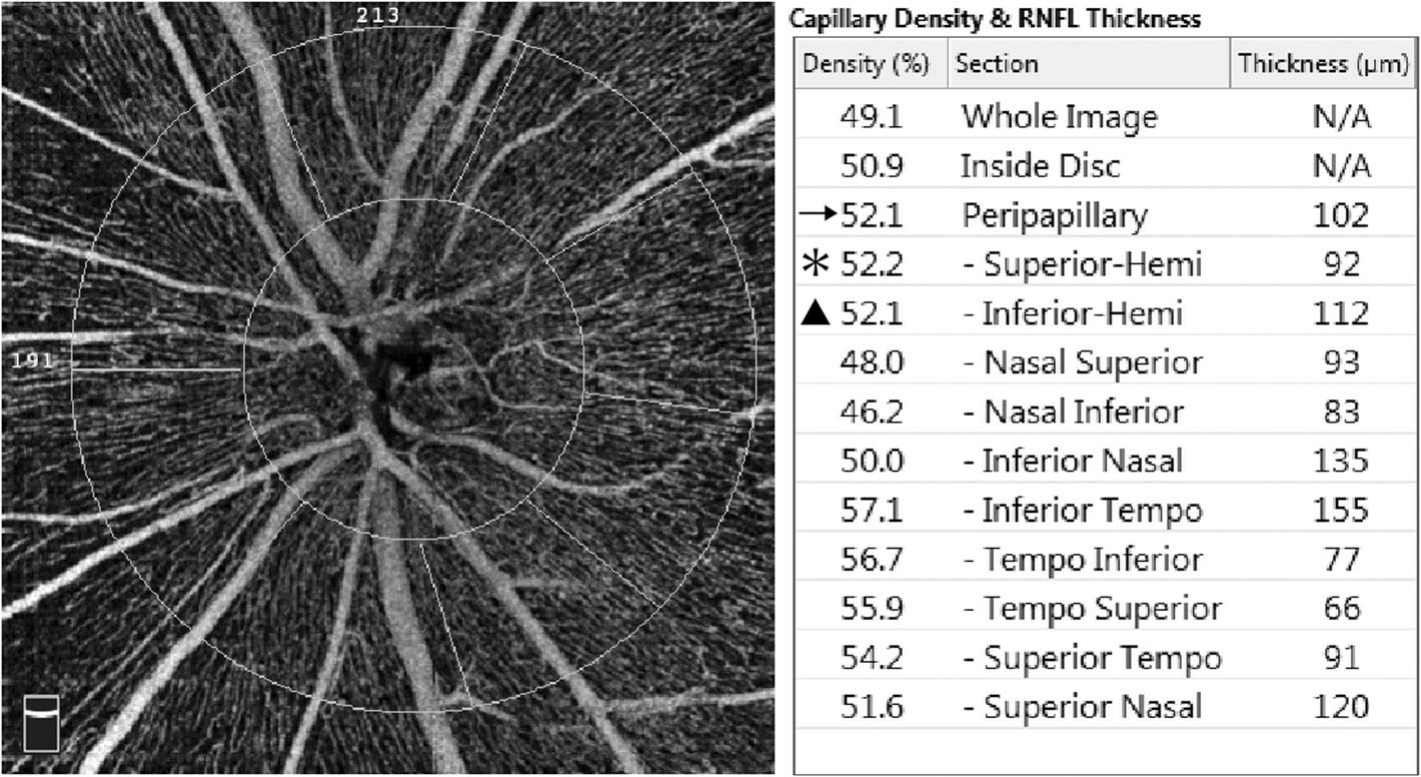
Measuring radial peripapillary capillaries (RPC) vessel density (VD) based on Garway-Heath’s map. (Left) The representative 4.5 mm × 4.5 mm en face OCT angiography image is centered on optic nerve head. The peripapillary area is overlaid by annular contour lines with 2 mm and 4 mm diameter around disc margin. The peripapillary area is further divided into eight sectors according to Garway-Heath’s map. (Right) The built-in software automatically calculates RPC VD based on Garway-Heath’s map. The global RPC VD (arrow) is measured from the 360-degree annular peripapillary area. The RPC VD of the superior (asterisk) and inferior (triangle) hemifield is measured from the superior four sectors and the inferior four sectors, respectively

For quantitative assessment of RPC VD, we used a built-in software AngioVue AngioAnalytics (Version 2017.1, Optovue Inc. Fremont, CA, USA). This software uses a split-spectrum amplitude decorrelation angiography algorithm with three-dimensional projection artifact removal technique and provides selective information on capillary VD in the RPC layer. By the definition of the software, peripapillary area is the area between the 2 mm and 4 mm diameter annular contour lines around disc margin. Manual adjustments of disc contour and layer segmentation were made in case of need. This software automatically generated the RPC VD of the global 360-degree area, the superior hemifield, and the inferior hemifield, based on Garway-Heath’s map (Fig. 1) [18]. The OCT angiography scan quality index (from 1 to 10) was also provided by the software.

### Outcome measures

Primary outcome was the change in global RPC VD from before SO removal to 3 months after SO removal, compared with the contralateral eyes.

Secondary outcomes included: 1) the change in RPC VD at 3 months after SO removal in superior and inferior hemifield, compared with the contralateral eyes; 2) the trend of changes in global RPC VD before SO removal and at 10 days, 1 month, and 3 months after SO removal; and 3) the association between global RPC VD change and age, sex, AL, preoperative macula status, combined cataract surgery with SO removal, SO tamponade duration, as well as full SO tamponade.

We conducted sensitivity analysis by comparing the change in global RPC VD between affected eyes and contralateral eyes in a subgroup of patients who did not have cataract surgery during SO removal, to eliminate the confounding effect from signal strength improvements due to cataract removal.

### Statistical analysis

We analyzed data using SPSS program version 22.0 (IBM, Inc., Chicago, IL). Kolmogrov–Smirnov method was used for test of normality. In the comparison of demographic and clinical characteristics between affected and contralateral eyes, we used Wilcoxon signed ranks test and Fisher exact test for continuous and dichotomous variables, respectively. In the comparison of RPC VD changes between affected and contralateral eyes, we used mixed effect model. We evaluated the main effect of SO removal surgery on RPC VD change, with the changes in IOP and OCT angiography scan quality index adjusted as covariates. The trend of changes in RPC VD after SO removal was analyzed using repeated measures ANOVA (analysis of variance) and Bonferroni’s post hoc test. Pearson or Spearman correlation analysis, where appropriate, was used to study RPC VD change for its association with demographic and clinical characteristics. A *P* value of less than 0.05 was considered statistically significant.

## Results

### Demographic and clinical characteristics

The study enrolled 31 patients. After excluding 9 patients (3 lost to follow-up after SO removal, 3 had poor OCT angiography image quality before SO removal due to severe cataract, 2 had internal limiting membrane peeling for macular hole repairment or subfoveal residual perfluorocarbon removal, and 1 had scleral buckling for recurrent retinal detachment), we included 22 patients for analysis. The average duration of SO tamponade was 101.3 days (range, 90 to 119 days). The demographic and clinical characteristics are summarized in Table 1.

**Table 1:** Demographic and Clinical Characteristics (*n* = 22)

Characteristics	Affected eye	Contralateral eye	*P* value^*^
Age (Years)	52.2 ± 12.3		–
Sex (Male/Female)	15/7		–
Refractive Error (Diopters)	−2.5 ± 2.3	−2.4 ± 2.3	0.72
Axial Length (mm)	24.81 ± 1.24	24.79 ± 1.29	0.86
Macula Status (On/Off)	3/19	NA	NA
SO Removal Combined with Cataract Surgery (Yes/No)	9/13	NA	NA
SO Tamponade Duration (Days)	101.3 ± 8.4	NA	NA
Full SO Tamponade^a^ (Yes/No)	15/7	NA	NA

Age, refractive error, and axial length are shown as mean ± standard deviation

^*^P values are from Wilcoxon signed ranks test for continuous variables

^a^Silicone oil tamponade is deemed as full if oil-water interface is not visible under indirect ophthalmoscope after pupil dilation

*SO* silicone oil, *NA* not applicable

### Peripapillary vessel density

In the affected eyes, the scan quality index before and 90 days after SO removal was 7.2 ± 1.0 and 7.8 ± 1.0, respectively. The mean change in scan quality index was 0.6 (95% CI, − 0.1 to 1.3, *P* = 0.11). The scan quality index in the contralateral control eyes did not change after SO removal (8.1 ± 1.0 versus 8.1 ± 1.2, *P* = 0.80). The global RPC VD increased after SO removal (Table 2). The average change in global RPC VD was 1.3% (0.3 to 2.3%) and − 0.4% (95%CI, − 1.4 to 0.7%) in affected and contralateral eyes, respectively. Mixed effect model analysis revealed that the difference in RPC VD change between affected and contralateral eyes was 1.7% (95%CI, 0.4 to 3.0%), which was statistically significant (*P* = 0.007) after adjusting for the changes in IOP and OCT angiography scan quality index.

**Table 2:** Comparison of Peripapillary Vessel Density Changes After Silicone Oil Removal Between Affected and Contralateral Eyes (*n* = 22)

Measurements		Affected eyes	Contralateral eyes
Global RPC VD	Before SOR	46.8 ± 3.7%	51.0 ± 3.3%
	After SOR	48.1 ± 3.8%	50.6 ± 3.4%
	Change	1.3% (0.3 to 2.3%)	− 0.4% (− 1.4 to 0.7%)
	Difference of change^a^	1.9% (0.6 to 3.2%), *P* = 0.007	
Superior RPC VD	Before SOR	46.8 ± 3.9%	51.1 ± 3.8%
	After SOR	48.4 ± 3.6%	50.8 ± 3.8%
	Change	1.6% (0.6 to 2.7%)	−0.4% (−1.4 to 0.7%)
	Difference of change^a^	2.2% (0.9 to 3.4%), *P* = 0.002	
Inferior RPC VD	Before SOR	46.7 ± 4.3%	50.8 ± 3.2%
	After SOR	47.7 ± 4.4%	50.4 ± 3.5%
	Change	1.0% (−0.2 to 2.1%)	−0.4% (−1.5 to 0.7%)
	Difference of change^a^	1.6% (−0.0 to 3.2%), *P* = 0.06	

Vessel densities before and after silicone oil removal are described as mean ± SD

Changes in vessel densities are described as mean (95% confidence interval)

^a^Difference of change in vessel densities between affected and contralateral eyes was analyzed using mixed model analysis and presented as mean (95% confidence interval). The *P* value represents the main effect of SOR with changes in intraocular pressure and OCT angiography image quality score adjusted as covariates

*RPC* Radial Peripapillary Capillaries, *VD* Vessel Density, *SOR* Silicone Oil Removal

The increase of RPC VD after SO removal mainly occurred in the superior hemifield, which was 1.6% (95%CI, 0.6 to 2.7%). The increase of RPC VD in the inferior hemifield was 1.0% (95%CI, − 0.2 to 2.1%) after SO removal (Table 2).

**Fig. 2:**
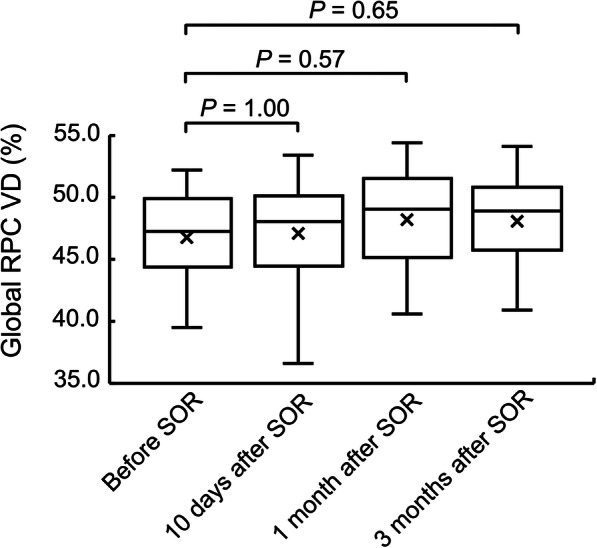
The whisker plot of global radial peripapillary capillaries (RPC) vessel density (VD) measured before and at 10 days, 1 month and 3 months after silicone oil removal (SOR) in patients who underwent pars plana vitrectomy and silicone oil tamponade for rhegmatogenous retinal detachment repair. The mean, median, and 25th to 75th percentile of RPC VD is shown by cross, horizontal line, and box, respectively. An increase in peripapillary vessel density after SOR is revealed by repeated measures ANOVA (*F* = 5.417, *P* = 0.002). The results of Bonferroni’s post hoc test are indicated by *P* values above horizontal bars

There was a significant trend of increase in global RPC VD after SO removal (*F* = 5.417, *P* = 0.002) (Fig. 2).

No association was found between the change in global RPC VD and age (*P* = 0.61), sex (*P* = 0.71), refractive error (*P* = 0.87), axial length (*P* = 0.51), preoperative macular status (*P* = 0.38), combined cataract surgery during SO removal (*P* = 1.00), duration of SO tamponade (*P* = 0.63), or full SO tamponade (*P* = 0.76).

In 13 patients who did not have cataract surgery during SO removal, the scan quality index in the affected eyes before and 90 days after SO removal was 7.3 ± 1.1 and 7.9 ± 0.9, respectively. The mean change in scan quality index was 0.5 (95% CI, − 0.5 to 1.5, *P* = 0.27). The scan quality index in the contralateral control eyes did not change after SO removal (7.9 ± 1.1 versus 8.0 ± 1.2, *P* = 0.34). In the affected eyes, the global RPC VD increased by 1.4% (95%CI, 0.2 to 2.5%) after SO removal, although mixed effect model revealed no statistically significant difference (*P* = 0.08) compared with the − 0.0% (95%CI, − 1.4 to 1.3%) change in contralateral eyes.

## Discussion

To our knowledge, the current study is the first one that investigated the effect of SO on peripapillary blood flow. We found a mild but definite increase of RPC VD in SO filled eyes following SO removal. Our results suggested that SO may have potential negative effect on microvascular blood flow around optic nerve head.

The increase in RPC VD reflected a mild but true increase in microvascular blood flow around optic nerve head after SO removal. The repeatability of OCT angiography measurements had been well demonstrated in previous studies [19, 20]. Factors that could influence the accuracy of OCT angiography measurements included IOP fluctuation [21], refractive media opacity and insufficient signal strength [20, 22, 23], diurnal variations and blood pressure changes [24]. In our study, we made several efforts to limit confounding factors. First, with healthy fellow eyes serving as controls, the impact of systemic errors, such as diurnal variations and systemic circulatory changes, could be minimized. Second, the main outcome measures were acquired at least 3 months after primary vitrectomy and 3 months after SO removal, which was a long enough period for acute surgical inflammation and IOP fluctuations to subside. Thus, the resulting RPC VD measurements were unlikely to be affected by postoperative corneal edema, anterior chamber flares, hypotonic papilledema, or potentially other unusual conditions. Third, we excluded subjects with severe cataract before SO removal and included only patients with adequate OCT angiography image quality. Also, the sensitivity analysis in patients who did not have cataract surgery during SO removal detected similar increase in RPC VD. Therefore, the increase in peripapillary vessel density was unlikely to be confounded by improvement in optic media clarity due to cataract removal. Fourth, although OCT angiography scan quality index exhibited mild increase after SO removal, its changes were adjusted as covariates when comparing RPC VD changes between affected eyes and controls. Fifth, trend analysis revealed that RPC VD after SO removal manifested a steady increase rather than an irregular fluctuation, supporting the reliability of our results. One might argue that the recovery of peripapillary vessel density could reflect a continuous improvement of optic nerve microcirculation after retinal reattachment. However, peripapillary vessel density increase was not observed from 1 month to 3 months after primary vitrectomy surgery (46.7 ± 4.0% versus 46.8 ± 3.7%, *P* = 0.92, paired *t* test), suggesting that the increase of RPC VD after SO removal was not part of a prolonged recovery process from retinal reattachment. One might also argue that SO tamponade and refractive changes after SO removal will affect image magnification which will further influence RPC VD measurements. By comparing the disk diameter on en-face OCT images before and after SO removal (1.518 ± 0.226 mm and 1.518 ± 0.229 mm, *P* = 1.00), we did not detect significant difference in image magnification with and without SO tamponade. Therefore, SO tamponade and refractive changes are unlikely to significantly influence RPC VD measurements. Others might argue that OCT angiography images under SO could be dark because of masking artifact. However, high quality images acquisition could be achieved in SO filled eyes when a suitable angle of scan could be found to avoid silicone oil reflection. Based on the above considerations, the increase in RPC VD was likely to be secondary to SO removal instead of other confounders. Although the amount of RPC VD increase was small, we are confident that a real improvement of peripapillary capillary density occurred after the removal of SO.

The superior hemifield of RPC showed a more prominent increase in VD than the inferior hemifield, suggesting that SO might affect the superior peripapillary microvasculatures more than the inferior vessels. One possible explanation was that the buoyancy of silicone oil exerted compression on peripapillary capillaries. The specific gravity of standard SO is lower than intraocular fluid. This physical property allows for a strong ability to tamponade the superior peripheral retina, while the lower periphery may not be efficiently supported. Although SO can support the area around optic disc and the larger posterior pole, we postulate that the superior hemifield of RPC might have received greater compression from SO tamponade. Interestingly, unlike RPC VD, the global RNFL thickness change at 3 months after SO removal did not show significant difference between affected and contralateral eyes (95%CI, − 3.1 μm to 6.7 μm, *P* = 0.47, mixed effect model). This indicated that the retinal nerve fibers might not be as vulnerable to mechanical compression as peripapillary capillaries were.

We tried to examine global RPC VD change after SO removal for its association with demographic and clinical characteristics including duration of SO tamponade and full SO tamponade, but none of these factors was associated with RPC VD change. Several reasons might account for the negative findings. First, the judgement of full SO tamponade relied on not only the quantity of SO in eye, but also the skill of examiner and the diameter of dilated pupil. Second, duration of SO tamponade was 3 to 4 months according to our institute’s common practice. If SO tamponade was kept for a longer duration, we might be able to detect a positive correlation between RPC VD change and SO tamponade duration. According to previous studies, longer duration of SO tamponade might be related with retinal structural abnormalities [25]. Third, the small sample size might be insufficient to yield significant results in correlation analysis.

In the current study, we did not evaluate RPC VD changes before and after primary vitrectomy and SO tamponade. Several difficulties existed in such investigation. First, most RRD patients at presentation had low vision, poor fixation, and considerable vitreous opacity which reduced the quality OCT angiography images. In some patients, the peripapillary retina was even detached, resulting in segmentation errors in OCT angiography images which could hardly be fully adjusted. Therefore, the accurate evaluation of RPC VD before primary vitrectomy and SO tamponade was difficult. Second, to evaluate the solitary effect of SO tamponade on peripapillary vessel density and eliminate the confounding effect from surgical trauma of primary vitrectomy, a group of controls who underwent vitrectomy without SO tamponade would be needed. However, legal SF6 or C3F8 product for medical use was unavailable across China until September 2019 when we end the current study. Considering the above difficulties, we studied the effect of SO on peripapillary vessel density by comparing RPC VD before and after SO removal, based on the assumption that SO removal without retinal manipulations caused limited surgical trauma and minimal pathophysiological changes in the posterior segment of eye. Previous studies had compared the effect of different types of tamponade on retinal structures using OCT. A case series by Christensen et al. [7] performed OCT scans in RRD patients and detected inner retinal thinning in SO filled eyes compared with gas filled eyes. On the contrary, a case control study by Bansal et al. [26] suggested that optic neuropathy after vitrectomy was related with intraoperative systemic hypotension and low ocular perfusion, but not related with the type of tamponade. Further studies are needed to compare peripapillary vessel density changes in eyes with SO and gas tamponade.

The strengths of this study included prospective design, longitudinal follow-up, and high-quality data acquisition. We acknowledge several limitations in our study. First, we excluded patients with high myopia whose peripapillary microvasculatures might react differently to the use of SO. Second, our findings should be limited to RRD. We should be careful about generalizing to other vitreoretinal diseases. Third, the duration of SO tamponade was short, and the effect of long-term SO tamponade on peripapillary vessel density remained unknown. Fourth, we did not compare the effect of SO and gas tamponade on RPC VD changes, as was discussed above. Future studies should further examine this issue. Fifth, we only identified subclinical increase in peripapillary vessel density after SO removal, and the increase was not analyzed for its visual correlation.

## Conclusions

In patients who underwent vitrectomy and SO tamponade for RRD repair, we detected a mild increase in peripapillary capillary vessel density after SO removal, mainly in the superior hemifield of peripapillary area. Our findings suggested that SO removal was associated with a recovery of peripapillary microvascular blood flow, possibly due to the release of vascular compression. Our results implied that the use of SO should be held with caution due to its potential negative impact on optic nerve microcirculation, even when the duration of SO tamponade does not exceed four months. Our study also added new insights to the complex biologic mechanisms of SO related optic neuropathy.

## Data Availability

The datasets used and/or analysed during the current study are available from the corresponding author on reasonable request.

## Abbreviations

AL: Axial length
ANOVA: Analysis of variance
IOP: Intraocular pressure
NA: Not applicable
OCT: Optical coherence tomography
RPC: Radial peripapillary capillaries
SO: Silicone oil
SOR: Silicone oil removal
VD: Vessel density
RNFL: Retinal nerve fiber layers
PPV: Pars plana vitrectomy
